# Successful Treatment of Suspected Cannabinoid Hyperemesis Syndrome Using Haloperidol in the Outpatient Setting

**DOI:** 10.1155/2016/3614053

**Published:** 2016-08-11

**Authors:** Jennifer L. Jones, Karen E. Abernathy

**Affiliations:** ^1^Departments of Psychiatry and Internal Medicine, Medical University of South Carolina, 67 President Street, Charleston, SC 29425, USA; ^2^Department of Medicine, Division of General Internal Medicine and Geriatrics, Medical University of South Carolina, 135 Rutledge Avenue, Charleston, SC 29425, USA

## Abstract

Chronic use of cannabis can result in a syndrome of hyperemesis characterized by cyclical vomiting without any other identifiable causes. Cannabinoid hyperemesis syndrome (CHS) is seldom responsive to traditional antiemetic therapies. Despite frequent nausea and vomiting, patients may be reluctant to discontinue use of cannabis. We report a case of severe, refractory CHS with complete resolution of nausea and vomiting after treatment with haloperidol in the outpatient setting. After review of the literature, we believe this is the first reported successful outpatient treatment of CHS and suggests a potential treatment for refractory patients.

## 1. Introduction

Cannabinoid hyperemesis syndrome is a condition of cyclic nausea, vomiting, and abdominal pain in chronic cannabis users without other identifiable etiologies [1]. CHS is also associated with compulsive showering in hot water, age younger than 50 years, morning predominance of symptoms, excessive use of cannabis (one or more times per week over at least one year), and cessation of symptoms with cannabis abstinence [2, 3]. CHS may be difficult to diagnose, especially as cyclic vomiting syndrome (CVS) and cannabis withdrawal syndrome (CWS) have a similar presentation, though with important differences. CVS is characterized by recurrent episodes of nausea and vomiting, and patients often have a compulsion for bathing in hot water [4]. However, CVS is typically associated with depression or anxiety, and many patients also experience migraines; patients also frequently lack the overuse of cannabis found in CHS [1, 3, 5]. CHS may also be confused with CWS, which is associated with nausea and vomiting after abrupt cessation of cannabis use. Patients presenting with CWS usually have other symptoms in addition to nausea, vomiting, or abdominal pain such as irritability, sleep disturbance, decreased appetite, or depressed mood [6]. This constellation of symptoms is not typically present in CHS, and patients do not abstain from cannabis [2, 3].

The prevalence of CHS has been increasing, which likely reflects both an increasing awareness of the condition and a rise in cannabis use in the United States over the past decade [7]. Although patients with CHS may report escalating cannabis usage, they are usually unaware that cannabis use is the cause of their symptoms [1]. Symptoms are generally refractory to conventional antiemetics [1]. In this context, we report a case of an 18-year-old patient with refractory nausea that was successfully treated with a short course of haloperidol in the outpatient setting.

## 2. Case

An 18-year-old woman presented to an outpatient clinic at a tertiary medical center for further evaluation and treatment of one-year history of refractory nausea, vomiting, and abdominal pain. Patient stated that her symptoms were worse in the morning and relieved only by smoking marijuana, which she had been increasingly doing for the past two years. Her emesis was consistently nonbloody and nonbilious. The frequency of her symptoms had been progressing over the previous few months, and at the time of presentation, she was having persistent nausea and vomiting throughout the day. She reported using cannabis 2-3 times per day on average.

The initial physical exam was unremarkable and her vital signs were within normal limits. She had a normal BMI and appeared nondistressed and nonanxious. Her heart was of regular rate and rhythm without appreciable murmurs. Her lungs were clear to auscultation. Her abdomen was soft, nontender, nondistended, and without appreciable organomegaly. Her skin was warm, dry, and without appreciable rashes or lesions.

Laboratory findings including a basic metabolic profile, liver function tests, and a complete blood count were normal. The patient had previously undergone a comprehensive workup including gastric emptying study, esophageal pH studies, and upper endoscopy without any abnormalities. Prior to clinic presentation, she had multiple urine drug screens that tested positive for cannabis while she was symptomatic. The patient had formerly tried a variety of antiemetic medications including ondansetron, promethazine, prochlorperazine, metoclopramide, lorazepam, and omeprazole. However, she denied that any of these medications had helped her symptoms with the exception of limited relief with metoclopramide (which was discontinued secondary to diarrhea).

Given the patient's prolonged heavy cannabis use (with refusal to discontinue), her chronic nausea and vomiting (with lack of other features consistent with CVS or CWS), and no other identifiable cause, the patient was diagnosed with CHS. Interestingly, she did not have slowed gastric emptying, which is frequently associated with cannabis use in CVS [8], and we were unable to find any studies regarding the prevalence of delayed gastric emptying in CHS. The patient's lack of symptom relief from multiple antiemetics prompted a review of the literature, which revealed prior case reports of resolution of CHS symptoms using haloperidol in the emergency department setting [9]. Although the patient was unwilling to discontinue cannabis, she agreed to initiate a trial of haloperidol 5 mg daily for symptom relief. At the next visit, the patient reported complete resolution of the previously refractory nausea, vomiting, and abdominal pain within one day of starting treatment. She denied any adverse effects from the haloperidol, and she self-discontinued treatment after three weeks without any recurrence of nausea, vomiting, or abdominal pain. She was subsequently lost to follow-up; thus we were unable to determine her long-term prognosis.

## 3. Discussion

The pathophysiology behind cannabis-induced hyperemesis is not completely understood. Both endogenous cannabinoids (anandamide and 2-arachidonoylglycerol) and exogenous cannabinoids are known to act on CB1 and CB2 G-protein coupled receptors, which are primarily located in the central nervous system and the periphery, respectively [5]. The most prevalent (and psychogenic) cannabinoid in marijuana is 9-tetrahydrocannabinol (9-THC), although it is not known whether it is 9-THC, a cannabidiol, or a cannabigerol (related chemicals found in cannabis) that is responsible for the hyperemetic properties of cannabis seen in some chronic users [5]. While dronabinol (a synthetic version of 9-THC) is approved for the treatment of chemotherapy-induced hyperemesis, case reports have also suggested that it is capable of inducing emesis [10]. Animal studies indicate that cannabidiol has a biphasic effect in rodents, with low doses attenuating vomiting, but high doses potentiating emesis [11, 12], which may explain the paradoxical effects of different cannabinoids. Wallace et al. have previously suggested that the duration of cannabinol use may differentially attenuate or induce emesis, although this remains to be investigated [13]. In animal studies, cannabinoids have been shown to dysregulate the hypothalamus which correlates with hypothermia [14]. However, as hypothermia is not consistently associated with nausea, it is unclear what connection the hypothalamic dysregulation has with induction of CHS symptoms.

Although the antiemetic mechanism of haloperidol has not been conclusively established, it is most likely related to blockade of the postsynaptic dopamine receptors in the brain. Dopamine antagonism has been shown to decrease nausea and vomiting by lessening the effect of dopamine on D2 receptors within the chemoreceptor trigger zone, thus decreasing input to the medullary vomiting center [15]. This may explain its beneficial effect of reducing nausea and vomiting in our patient.

From a public health perspective, 22.2 million persons aged 12 or older in the United States were current users of cannabis in 2014, and this number continues to rise each year since 2002 according to the National Survey on Drug Use and Health [7]. At present, there are no reliably effective treatment regimens for patients with CHS who refuse to discontinue cannabis use, including conventional antiemetics. Pharmacologic studies indicate that haloperidol may act indirectly on CB1 receptors to relieve nausea, vomiting, and abdominal pain. Our findings in this case study are consistent with case reports from the emergency department setting [9] and suggest the need for further studies to evaluate the efficacy of haloperidol in the treatment of refractory CHS.
